# Exploring the Orthogonal Chemoselectivity of 2,4,6-Trichloro-1,3,5-Triazine (TCT) as a Trifunctional Linker With Different Nucleophiles: Rules of the Game

**DOI:** 10.3389/fchem.2018.00516

**Published:** 2018-11-01

**Authors:** Anamika Sharma, Ayman El-Faham, Beatriz G. de la Torre, Fernando Albericio

**Affiliations:** ^1^KRISP, College of Health Sciences, University of KwaZulu-Natal, Durban, South Africa; ^2^School of Chemistry and Physics, University of KwaZulu-Natal, Durban, South Africa; ^3^Department of Chemistry, College of Science, King Saud University, Riyadh, Saudi Arabia; ^4^Department of Chemistry, Faculty of Science, Alexandria University, Alexandria, Egypt; ^5^Department of Organic Chemistry, University of Barcelona, Barcelona, Spain; ^6^CIBER-BBN, Networking Centre on Bioengineering, Biomaterials and Nanomedicine, Barcelona Science Park, Barcelona, Spain

**Keywords:** *s*-triazine, triorthogonal linker, density functional theory, NBO calculations, electrostatic potential maps

## Abstract

The study involves exploring the three orthogonal sites for aromatic nucleophilic substitution in cyanuric chloride (TCT). The preferential order of incorporation of different nucleophiles (such as alcohol, thiol, and amine) was addressed both experimentally and theoretically. The preferential order for incorporating nucleophiles in TCT was found to be alcohol > thiol > amine.

## Introduction

**Figure 1:**
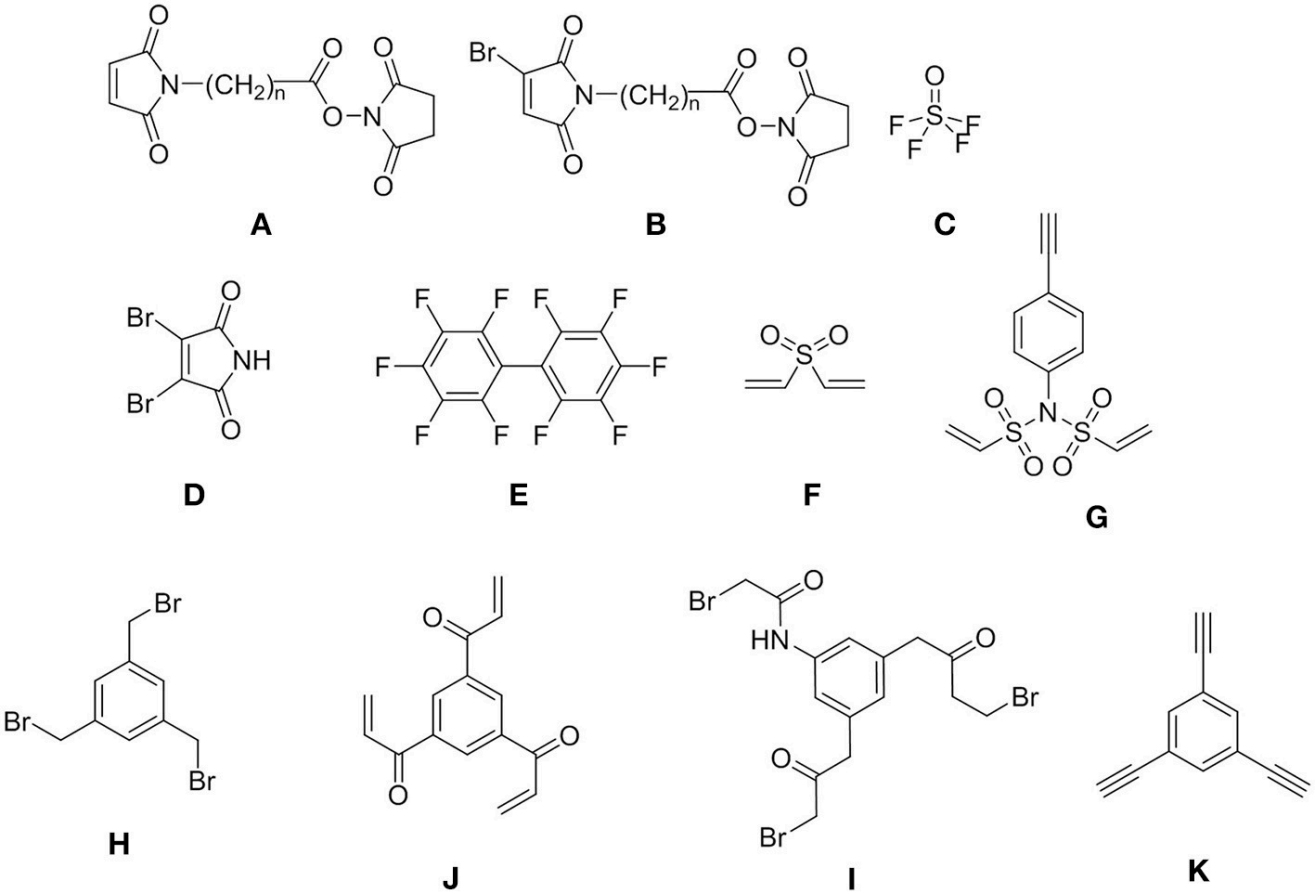
Examples of the most common linkers.

With the maturity of the disciplines such as chemical biology or nanobiotechnology and their translation into the concepts of new drugs, there is an increasing need for strategies to link chemical/biological moieties to render chimeras, polyligands, conjugates (intermolecularly) or cycles (intramolecularly). The cornerstone for the preparation of these new molecular structures is the *LINKER* (Wills and Balasubramanian, 2003; Gil and Bräse, 2004). The molecule used for this purpose should enhance its own binding properties and should not jeopardize the biological activity of the new structure. The optimal property of the “naked” linker is the presence of two or more reactive functions, which allow assembly of the chemical/biological moieties in relatively mild conditions. However, the linker should ensure the stability of the new molecular structure and in some cases it should also facilitate the release of the cargo once the biological target has been reached (Gil and Bräse, 2004; Böhme and Beck-Sickinger, 2015). Recent years have witnessed an explosive development of linkers and related strategies. Although not an exhaustive list, Figure 1 shows several widely used linkers.

The most widely used linker is maleimide-acyl succinimide ester **(A)**, (Beck et al., 2017) which reacts first with amine to afford amide, followed by thiols through a Michael addition to yield sulfide. However, this ester **(A)** is highly susceptible to retro-Michael reactions with the consequent deconjugation, which liberates the drug before the target is reached (Tumey et al., 2014; Wei et al., 2016). Our group recently introduced a third functionality in the maleiimido-acyl sucinimido ester system that allows the introduction of a third molecule through nucleophilic substitution on the Br (Ramesh et al., 2016). With the same idea of trifunctionality, Sharpless and co-workers (Li S. et al., 2017) described the use of thionyl tetrafluoride **(C)**, which allows the installation of three molecules onto the linker. Although symmetrical, linkers **D** (Youziel et al., 2014) and **E** (Fadzen et al., 2017) allow sequential introduction of two distinct compounds. This occurs because the reactivity of the second symmetrical halogen changes once the first halogen has been substituted. Pentelute developed a series of linkers based on decafluoro(bi)phenyl derivatives (Lautrette et al., 2016). The rest of the linkers are completely symmetrical in structure and reactivity, thereby hampering the introduction of three distinct molecules. The divinyl sulfone **(F)** allows the double incorporation of N, O, S nucleophiles (Houen and Jensen, 1995). Another interesting linker is the *N*-phenyl-divinylsulfonamide **(G)** (Li Z. et al., 2017) with C_2v_ symmetry, which, in addition to the double substitution through a Michael addition, also allows the introduction of another molecule due to the presence of a reactive group in the phenyl ring. Other trifunctional linkers **H, I** and **J** with D_3h_ symmetry have also been extensively used by Heinis and co-workers (Deyle et al., 2017) and Timmerman and co-workers for the synthesis of byciclic peptides (Timmerman, 2012). As of yet, 2,4,6-trichloro-1,3,5-triazine (cyanuric chloride, TCT)^1^, which also has D_3h_ symmetry, has not been explored.

Given the efficient reactivity of TCT with a variety of nucleophiles, it is frequently used as an organic synthetic building block or template to access complex molecular architectures (Blotny, 2006; Rapoport and Smolin, 2009). TCT is an attractive linker due to its low cost, commercial availability, and ease of stepwise substitution of the three chlorine atoms (Blotny, 2006). TCT derivatives are known to exhibit a broad range of biological activities, including anti-bacterial (Solankee et al., 2010; Gavade et al., 2012; Patel et al., 2012), anti-fungal (Singh et al., 2012), anti-cancer (Brzozowski and Saczewski, 2002; Zhu et al., 2012; Kumar et al., 2013), anti-malarial (Kumar et al., 2009; Sunduru et al., 2010), anti-TB (Sunduru et al., 2010; Avupati et al., 2013), as well as herbicidal effects (Zhao et al., 2011) etc. Several research groups have also explored the application of TCT in solid phase synthesis by means of a combinatorial approach (Masala and Taddei, 1999) for the synthesis of dendrimers (Simanek et al., 2009, 2013; Lim et al., 2012) and non-covalently bound supra-molecular aggregates (Lehn et al., 1990; Mathias et al., 1994; Timmerman et al., 1997; Timmerman and Prins, 2001).

The relative weaker resonance energy of TCT provides reactivity at three distinct points for aromatic nucleophilic substitution using a one-pot procedure (Blotny, 2006). The uniqueness of TCT is that it reacts with almost all types of nucleophiles (S, O, N). Furthermore, the three reactive points are connected as after the introduction of first nucleophile the reactivity of the other remaining Cl changes. Once the second substitution has taken place, the reactivity of the third Cl differs to that of the first two.

**Figure 2:**
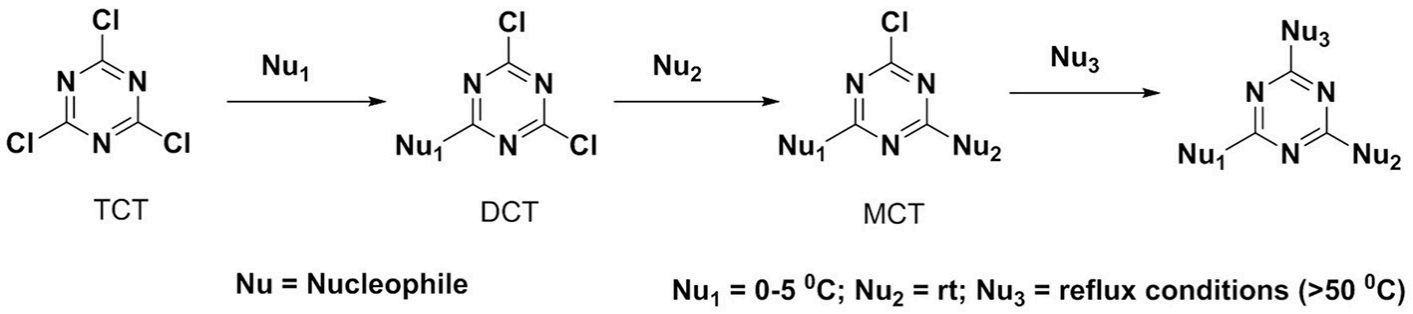
Reactivity of TCT *via* sequential nucleophilic substitution.

However, to the best of our knowledge, the reactivity of TCT toward distinct nucleophiles, including the three most important biologically ones (S, O, N) has not been addressed to date nor the effect of “-Cl” substitution with electron-donating groups (generated from nucleophiles) on the reactivity of TCT has been explored. It is known that the basic character of the incoming nucleophile that replaces the third chlorine is weakened due to the present substituent's in TCT, of the triazine ring through loss of σ-bond electron withdrawal of a chlorine atom and gain of π-orbital electronic donation of the added nucleophiles (Rapoport and Smolin, 2009). Thus, the incorporation of the first nucleophile into TCT can be performed at 0–5°C, while the second one requires room temperature, and the third one requires heating and even reflux conditions as shown in Figure 2 (Blotny, 2006).

Herein we discuss the unique properties of TCT as a linker that encompasses two key chemical concepts, *orthogonality* and *chemoselectivity*. In literature, the concepts of *orthogonality* and *chemoselectivity* are often used interchangeably (Wong and Zimmerman, 2013), and orthogonality has been considered as a subset of chemoselectivity (Wong and Zimmerman, 2013). However, the two concepts are complementary, thus providing the opportunity to converge into a new concept, namely *orthogonal chemoselectivity*.

**Figure 3:**
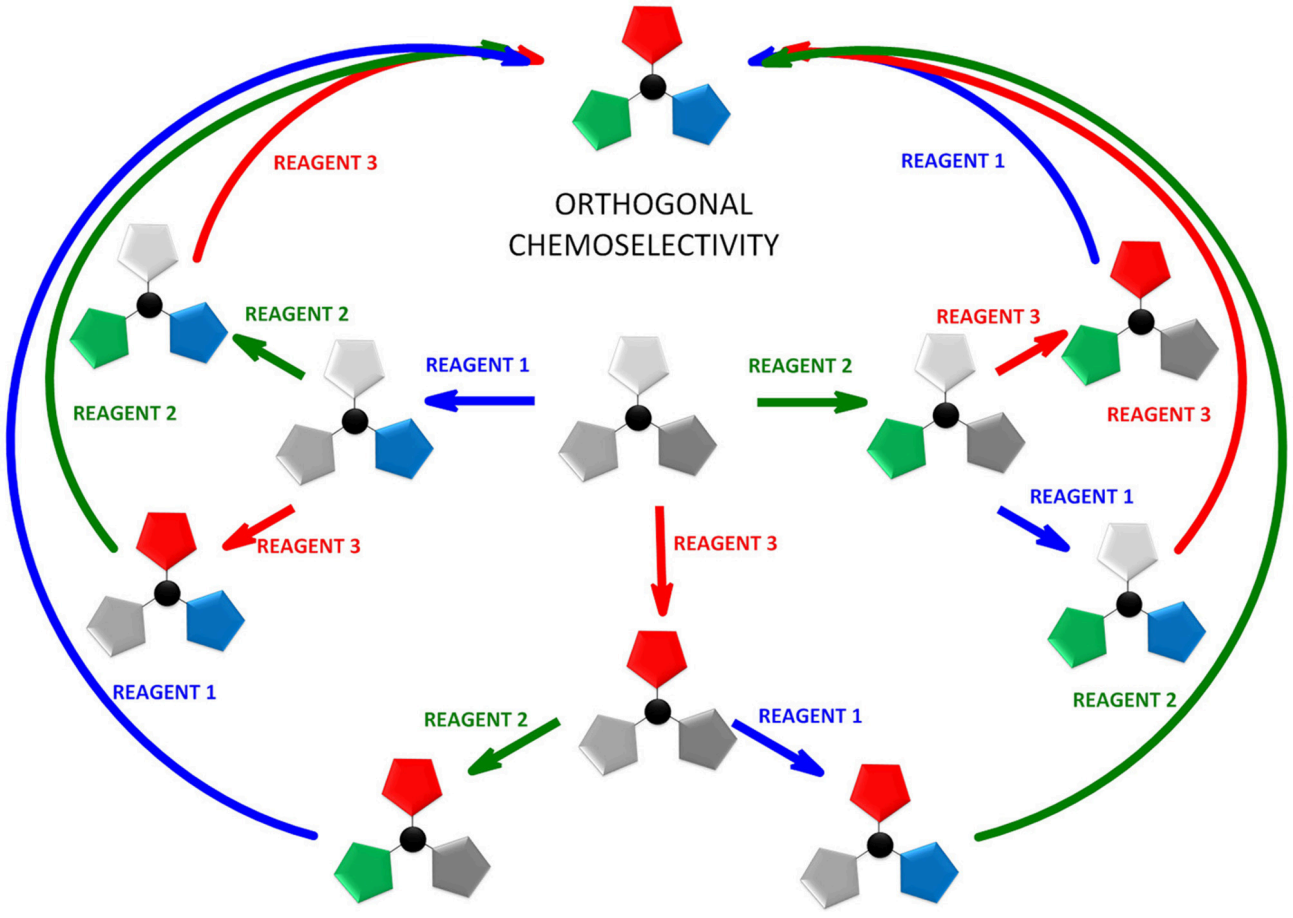
Sequential triorthogonal transformations of two functional groups.

The idea of *orthogonality* was first introduced in 1977 by Barany and Merrifield (1977), and then demonstrated by Barany and Albericio (1985), when it was applied to protecting groups. Thus, “orthogonal protecting groups” are those that can be successfully removed by different chemical mechanisms in any order and in the presence of other protecting groups. In 1983, Trost (1983) introduced the concept of *chemoselectivity* as the ability to discriminate between reactive sites. The fusion of these two concepts should allow “discrimination between reactive sites in any order.” Here we explored the scope and limitation of *orthogonal chemoselectivity* applied to TCT (Figure 3), both experimentally and theoretically.

## Experimental

### Materials and methods

2,4,6-Trichloro-1,3,5-triazine (cyanuric chloride, TCT), sec-butyl amine, 3-methyl-2-butanthiol and 2-phenyl ethanol, and diisopropylethylamine were purchased from Sigma-Aldrich (Sigma-Aldrich, Germany). The solvents used were of analytical and HPLC reagent grade. Magnetic resonance spectra (^1^H and ^13^C) were recorded with Bruker 400 MHz, and chemical shift values are reported in δ units (ppm) using TMS as internal standard. Follow-up of the reactions and checks of the purity of the compound were done by TLC on silica-gel-protected aluminum sheets 60 F254 (Merck), and the spots were detected by exposure to UV light at λ = 254 nm. Analytical HPLC was performed on an Agilent 1100 system using a Phenomenax C_18_ column (3 μm, 4.6 × 50 mm) by dissolving the sample in CH_3_CN only. Chemstation software was used for data processing. Buffer A: 0.1% TFA in H_2_O, buffer B: 0.1% TFA in CH_3_CN were used in HPLC. LCMS was performed on a Shimadzu 2020 UFLC using a YMC- Triart C_18_ (5 μm, 4.6 × 150 mm) column, and data processing was carried out using Lab Solution software. Buffer A: 0.1% formic acid in H_2_O, buffer B: 0.1% formic acid in CH_3_CN.

### Synthesis of 2,4-dichloro-6-substituted *s*-triazine (X-DCT)

TCT (50 mg, 0.27 mmol) was dissolved in DCM (1 mL) and cooled to 0°C for 5 min. Nucleophile (0.27 mmol) was then added to the above stirring solution, followed by addition of DIEA (47 μL, 0.27 mmol). The reaction was stirred at 0°C for 30 min. The progress of the reaction was monitored by TLC (ethyl acetate/hexane as mobile phase) until no starting material was observed. The reaction mixture was diluted with 5 mL of DCM and washed several times with water to remove DIEA salts. The organic layer was collected, dried over MgSO_4_, filtered and concentrated to afford pure product, which was used for the next step without further purification.

#### N-(sec-butyl)-4,6-dichloro-1,3,5-triazin-2-amine

Semi-solid; HPLC [5-95% of CH_3_CN (0.1% TFA/ H_2_O (0.1%TFA) over 15 min] *t*_*R*_ = 9.72 min; ^1^H NMR (400 MHz, CDCl_3_): 0.88 (t, *J* = 7.4 Hz, -CH_3_), 1.16 (d, *J* = 6.6 Hz, -CH_3_), 1.37 (m, -CH_2_), 1.45 (m, -CH), 5.81 (d, *J* = 6.2 Hz, NH); ^13^C NMR (100 MHz, CDCl_3_): 9.2, 18.9, 28.2, 48.1, 164.3, 168.7, 169.9.

#### 2,4-dichloro-6-((3-methylbutan-2-yl)thio)-1,3,5-triazine

Semi-solid; HPLC [5-95% of CH_3_CN (0.1% TFA/ H_2_O (0.1%TFA) over 15 min] *t*_*R*_ = 12.98 min; ^1^H NMR (400 MHz, CDCl_3_): 0.97 (d, *J* = 6.7 Hz, -CH_3_), 1.33 (d, *J* = 7.0 Hz, -CH_3_), 1.96 (m, -CH), 3.89 (m, -CH); ^13^C NMR (100 MHz, CDCl_3_): 16.4, 18.2, 18.4, 31.8, 47.0, 168.9, 185.7.

#### 2,4-dichloro-6-phenethoxy-1,3,5-triazine

Off-white solid; m.p. = 138–140°C; HPLC [5-95% of CH_3_CN (0.1% TFA/ H_2_O (0.1%TFA) over 15 min] *t*_*R*_ = 11.42 min; ^1^H NMR (400 MHz, CDCl_3_): 3.05 (t, *J* = 7.0 Hz, -CH_2_), 4.61 (t, *J* = 7.0 Hz, -CH_2_), 7.24 (m, ArH); ^13^C NMR (100 MHz, CDCl_3_): 34.8, 70.8, 127.0, 128.7,129.0, 136.6, 170.9, 172.5.

### Synthesis of 2-chloro-4,6-disubstituted *s*-triazine (XY-MCT)

Nucleophile (0.27 mmol) was added to DCT (0.27 mmol) in DCM (1 mL), followed by addition of DIEA (47 μL, 0.27 mmol). The reaction was stirred at r.t. for 12 h. The progress of the reaction was monitored by TLC (ethyl acetate/hexane as mobile phase) until no starting material was observed. The reaction mixture was diluted with 5 mL DCM and washed several times with water to remove DIEA salts. The organic layer was collected, dried over MgSO_4_, filtered and concentrated to afford pure product, which was used for the next step without further purification.

#### N-(sec-butyl)-4-chloro-6-((3-methylbutan-2-yl)thio)-1,3,5-triazin-2-amine

Semi-solid; HPLC [5–95% of CH_3_CN (0.1% TFA/ H_2_O (0.1%TFA) over 15 min] *t*_*R*_ = 13.48 min; ^1^H NMR (400 MHz, CDCl_3_): 0.87 (t, *J* = 7.5 Hz, -CH_3_), 0.93 (d, *J* = 7.0 Hz, -CH_3_), 1.14 (m, CH_3_), 1.27 (m, CH_3_), 1.49 (m, CH_2_), 1.94 (m, CH), 3.78 (m, -CH), 3.98 (m, -CH); ^13^C NMR (100 MHz, CDCl_3_): 9.2, 17.9, 18.6, 19.0, 19.2, 28.3, 31.7, 45.6, 47.6, 162.7, 181.3, 182.5.

#### N-(sec-butyl)-4-chloro-6-phenethoxy-1,3,5-triazin-2-amine

Semi-solid; HPLC [5-95% of CH_3_CN (0.1% TFA/ H_2_O (0.1%TFA) over 15 min] *t*_*R*_ = 12.08 min; ^1^H NMR (400 MHz, CDCl_3_): 0.86 (t, *J* = 7.4 Hz, -CH_3_), 1.12 (d, *J* = 6.6 Hz, -CH_3_), 1.47 (m, -CH_2_), 3.01 (t, *J* = 7.4 Hz, CH_2_), 3.98 (m, CH), 4.48 (t, *J* = 7.4 Hz, CH_2_), 7.22 (m, ArH); ^13^C NMR (100 MHz, CDCl_3_): 10.2, 20.1, 29.4, 35.1, 48.5, 68.7, 126.7, 128.6, 129.0, 137.4, 166.5, 170.4, 170.8.

#### 2-chloro-4-((3-methylbutan-2-yl)thio)-6-phenethoxy-1,3,5-triazine

Semi-solid; HPLC [5-95% of CH_3_CN (0.1% TFA/ H_2_O (0.1%TFA) over 15 min] *t*_*R*_ = 12.98 min; ^1^H NMR (400 MHz, CDCl_3_): 0.85 (d, *J* = 7.4 Hz, -CH_3_), 0.93 (d, *J* = 7.0 Hz, -CH_3_), 1.12 (d, *J* = 6.5 Hz, CH_3_), 1.92 (m, CH), 3.00 (m, CH_2_), 3.98 (m, CH_2_), 4.43 (m, CH), 7.22 (m, ArH); ^13^C NMR (100 MHz, CDCl_3_): 10.3, 29.6, 32.9, 35.3, 45.7, 47.8, 67.7, 126.6, 128.5, 129.0, 137.7, 165.5, 169.3, 182.1.

#### Synthesis of N-(sec-butyl)-4-((3-methylbutan-2-yl)thio)-6-phenethoxy-1,3,5-triazin-2-amine

Sec-butyl amine (0.27 mmol) was added to a stirring solution of 6 (0.27 mmol) in THF (1 mL), followed by addition of DIEA (0.27 mmol). The reaction mixture was heated to 75°C for 30 h. The progress of the reaction was monitored by TLC (ethyl acetate/hexane as mobile phase) until the complete consumption of starting material. Solvent was removed under vacuum, and the residue was dissolved in DCM (5 mL). The organic layer was washed several times with water to remove DIEA salts. The organic layer was collected, dried over MgSO_4_, filtered and concentrated to afford pure product (**7**).

Semi-solid; HPLC [5-95% of CH_3_CN (0.1% TFA/ H_2_O (0.1%TFA) over 15 min] *t*_*R*_ = 16.78 min; ^1^H NMR (400 MHz, CDCl_3_): 0.94 (m, -CH_3_), 1.28 (m, -CH_3_), 1.95 (m, CH_2_), 1.98 (m, -CH), 3.02 (t, *J* = 7.4 Hz, CH_2_), 3.80 (m, CH), 4.40 (t, *J* = 7.4 Hz, CH_2_), 7.22 (m, ArH); ^13^C NMR (100 MHz, CDCl_3_): 16.6, 16.7, 18.0, 18.6, 28.7, 31.8, 34.1, 45.3, 67.3, 73.1, 125.6, 127.5, 127.9, 136.4, 166.5, 178.3, 181.7.

### Theoretical calculations

The models were drawn using GaussView05, and all quantum chemical calculations were performed using Gaussian09 (Frisch et al., 2009) with B3LYP functional (Becke, 1988; Lee et al., 1988) and 6-311G++(d,p) basis set. No solvent corrections were made with these calculations. Vibration analysis showed that the optimized structure indeed represents a minimum on the potential energy surface (no negative eigenvalues).

## Results and discussion

### Chemistry

**Figure 4:**
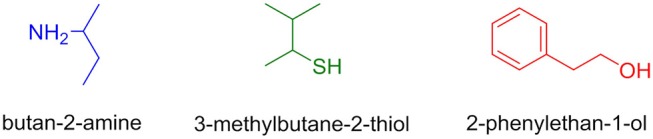
Nucleophiles selected for aromatic nucleophilic substitution on TCT.

Here we evaluated the incorporation of different nucleophiles (O, S, and N) that are commonly present as side chain functionalities of amino acids in biological systems (Bork et al., 2003; Lee et al., 2011; Chen et al., 2014). As per the ease of availability in our laboratory, the following three different nucleophiles (Figure 4) were used for the purpose of the study.

**Scheme 1:**
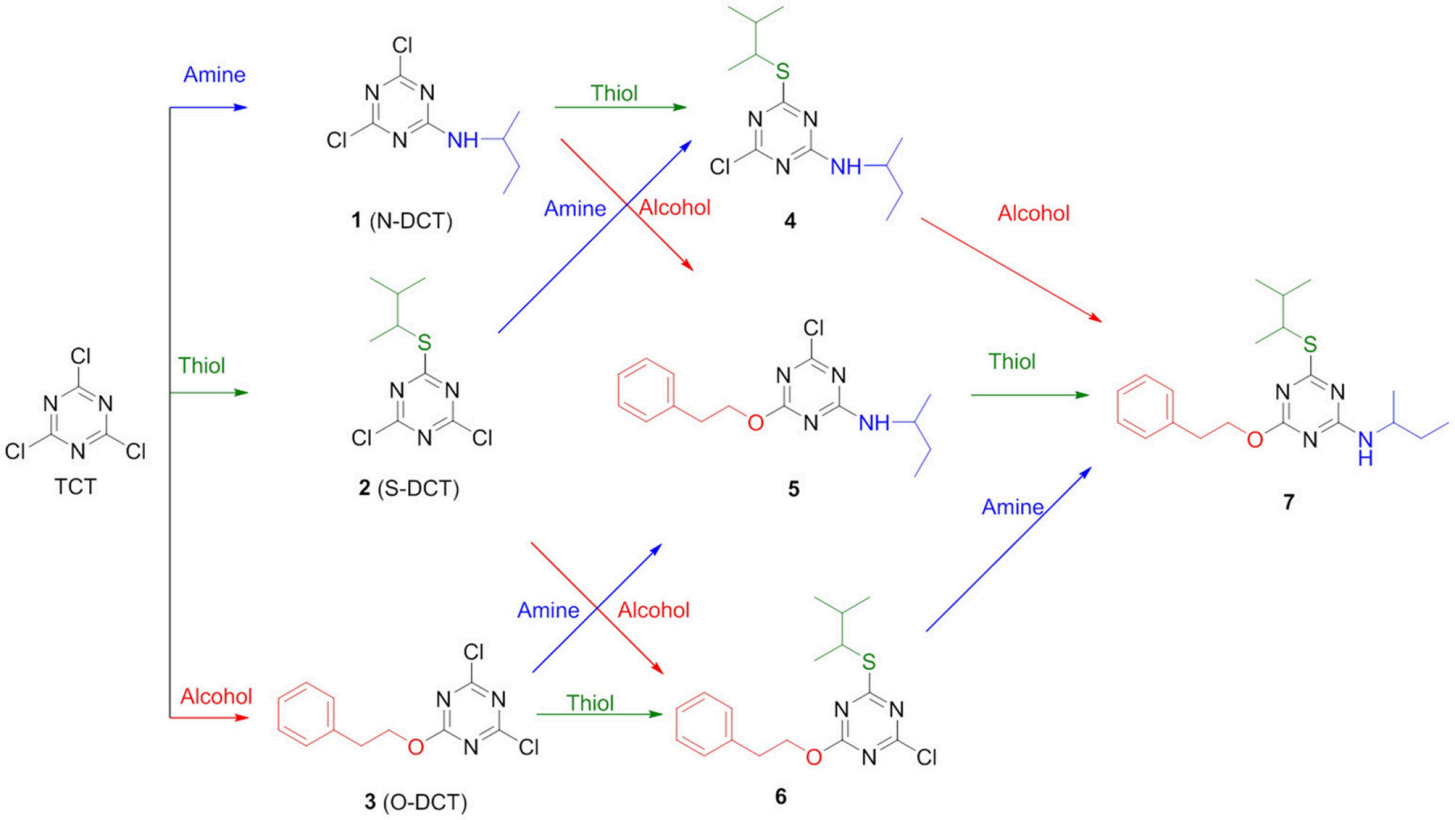
Different possible routes for the synthesis of **7**.

In order to determine the correct order of incorporation of distinct nucleophiles into TCT, an idea of their behavior is necessary. To the best of our knowledge, Pearson was the first to categorize nucleophiles into hard and soft (Pearson, 1963). Among the selected nucleophiles, butan-2-amine and 2-phenylethan-1-ol are considered hard nucleophiles as they are small, highly charged, and have a high charge/radius ratio. 3-Methylbutane-2-thiol is classified as soft nucleophile as it is larger, has lower electronegativity, and is polarizable (Pearson, 1963, 1997). Nucleophilicity is determined by charge, electronegativity, and solvent (Gazquez and Mendez, 1994). In the present study, we used distinct nucleophiles with the same solvent for comparison; hence, the charge factor and solvent factor can be ignored. The most crucial factor left for evaluation in this case is electronegativity (nucleophilicity). In fact, the electronegativity of O is higher than that of N and S (Speight, 2005). The main objective of this study was to evaluate what the optimal sequential route was for the incorporation of the nucleophiles O, S, and N on the TCT moiety to obtain compound **7** (Scheme 1).

**Figure 5:**
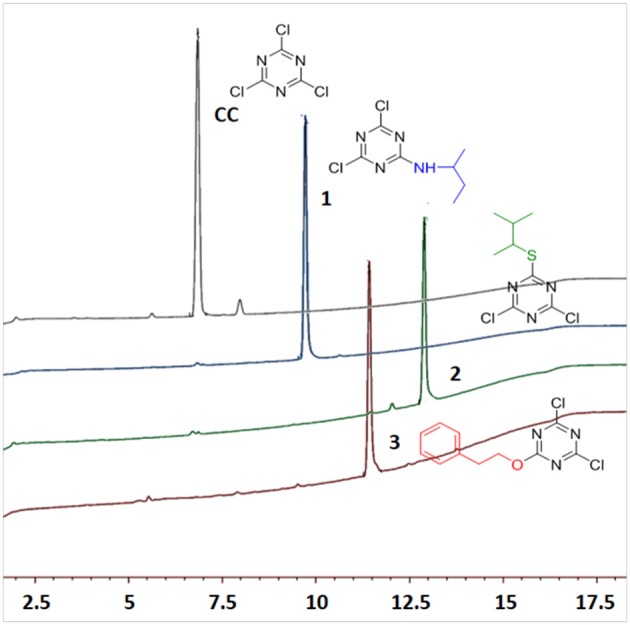
HPLC spectrum of **1**, **2**, and **3** [5–95% of CH_3_CN (0.1% TFA/H_2_O (0.1%TFA) over 15 min].

Several reports reveal that TCT undergoes nucleophilic substitution reaction in the presence of base (Rapoport and Smolin, 2009). Several attempts were therefore made to elucidate the common strategy to achieve the smooth replacement of first “Cl” in TCT. Parallel reactions were attempted with nucleophiles butan-2-amine, 3-methylbutane-2-thiol and 2-phenylethan-1-ol with TCT. As found in literature (Manohar et al., 2010; Rana et al., 2014; Jameel et al., 2017), K_2_CO_3_ was the base of choice, using acetone as solvent for successful replacement of the first “-Cl” in TCT with a nucleophile. The reaction was monitored by thin layer chromatography TLC, which showed the complete consumption of TCT in 30 min. However, the major problem encountered with K_2_CO_3_ as base was its solubility in solvents. This feature limits the application of an inorganic base. We then attempted in parallel a reaction using triethylamine (TEA) and *N*,*N*-diisopropylethylamine (DIEA) as base in tetrahydrofuran (THF) and dichloromethane (DCM) as solvent, respectively. The reaction showed similar kinetics as for K_2_CO_3_. Comparison between TEA and DIEA revealed that the latter had a higher boiling point, thus making it more efficient under reflux conditions at higher temperatures. The optimum reaction conditions for the three nucleophiles was found to be at 0°C using DIEA as base and DCM as solvent. In this regard, the reaction was completed within 30 min in all the cases, affording pure derivatives **1**, **2**, and **3**, as shown by TLC and HPLC. After completion of reaction, the reaction mixture was washed with water to remove DIEA salts. The organic phase was dried over MgSO_4_, filtered, and concentrated to afford almost pure 2,4-dichloro-6-substituted *s*-triazine (X-DCT) (Figure 5).

**Figure 6:**
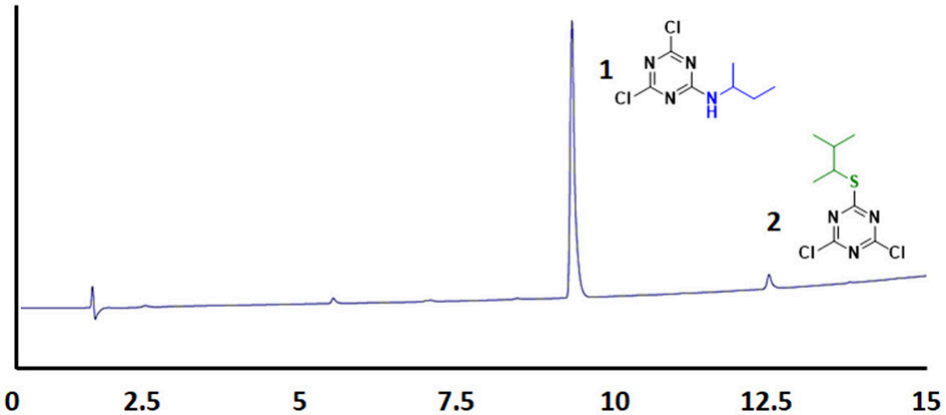
Competitive reaction of TCT with all nucleophiles in the same reaction.

Since all the nucleophiles undergo the nucleophilic substitution reaction resulting in the formation of **1**, **2**, and **3**, respectively, under similar reaction conditions, a competitive test was conducted to study the selectivity of the TCT in front of the three nucleophiles. In order to achieve this, one pot reaction was carried out at 0°C with TCT and all nucleophiles at the same time followed by addition of DIEA. The aim was to check the reactivity of amine, thiol and alcohol toward TCT under competitive reaction conditions. The reaction was monitored after 30 min with TLC which showed no presence of starting material. The crude sample was injected in HPLC (Figure 6). It is clearly evident that presence of amine is prevalent over thiol and alcohol when present in the same reaction mixture, since the major product formed is **1** (94%).

After the successful replacement of the first “-Cl,” attempt was made for the second nucleophilic substitution on the dichlorotriazine (X-DCT) derivatives for the synthesis of disubstituted monochloro-*s*-triazine (XY-MCT) derivative. Taking **1** (N-DCT) as substrate, attempts were made to react it with 3-methylbutane-2-thiol and 2-phenylethan-1-ol in the presence of DIEA in DCM at room temperature for 12 h to afford **4** and **5**, respectively. However, no product formation (neither **4** nor **5**) was observed, as determined by TLC and HPLC. This finding is consistent with earlier literature (Mori et al., 1982; Heeres et al., 2013) and could be attributable to the amine nucleophile deactivating the DCT core, thus impeding the reaction of a second nucleophile, 3-methylbutane-2-thiol and 2-phenylethan-1-ol. Reactions were attempted to synthesize 4 and 5 from 1 under heating conditions (75°C) using DIEA as base and DCM/Dioxane as solvent. However, no product formation was observed. This suggested the use of strong base (NaH) instead of DIEA or use of a conjugate base in place of the nucleophile. In both cases double substitution was observed along with the required products (as monitored by HPLC) maybe due to presence of strong reducing agent and conjugate base. In a similar manner, **2** (S-DCT) was reacted with butan-2-amine and 2-phenylethan-1-ol as nucleophile using DIEA as base and DCM as solvent at room temperature for 12 h. When butan-2-amine was the nucleophile, complete conversion of **2** to **4** (100%) was observed by HPLC and confirmed by LCMS. However, in the case of 2-phenylethan-1-ol, the reaction showed only 30% conversion of **2** (by HPLC and LCMS) to **4**. In order to achieve complete conversion, the reaction mixture was heated to 75°C using DIEA as base and THF as solvent. Under these conditions, the reaction showed 63% conversion to **6**, as determined by HPLC. Furthermore, **3** (O-DCT) was also made to react with butan-2-amine and 3-methylbutane-2-thiol, respectively, at room temperature for 12 h using DIEA as base and DCM as solvent. In both cases, 100% product formation was observed, as determined by TLC and HPLC. Both derivatives were worked up in a similar fashion as described above, affording good yields of relatively pure disubstituted mono chlorotriazine (XY-MCT) derivatives **5** and **6**, as determined by NMR.

**Figure 7:**
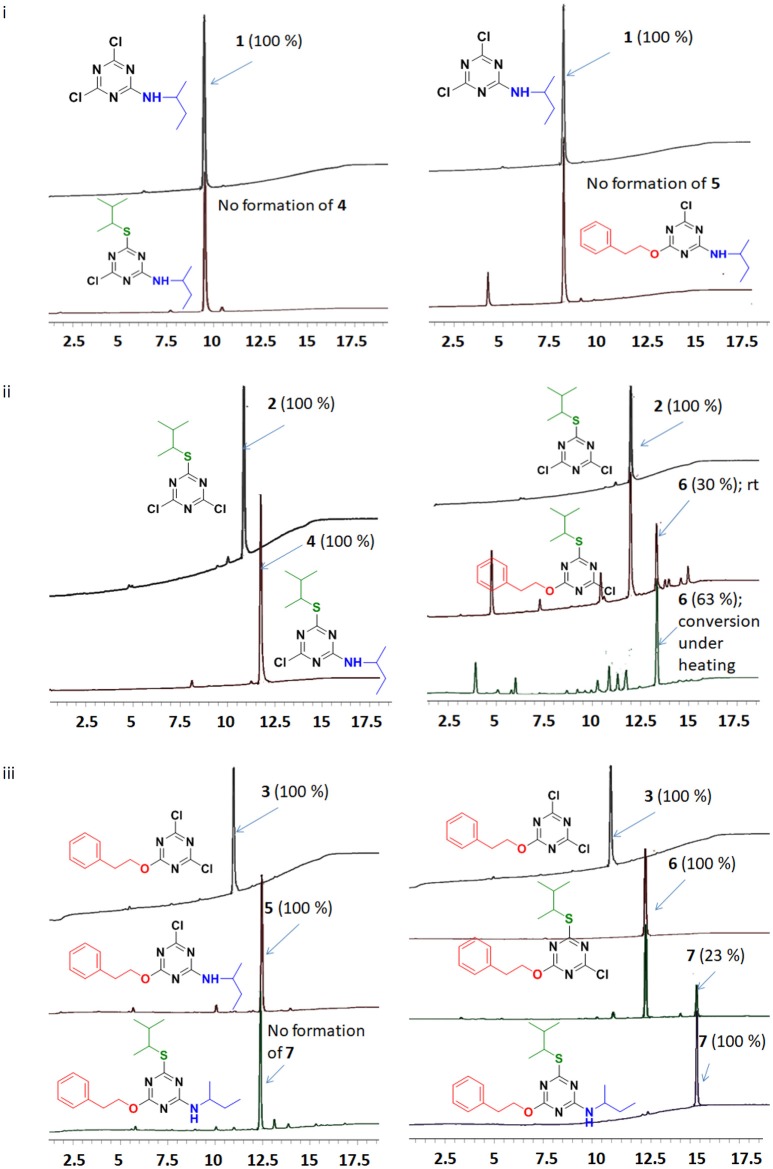
Reaction monitoring by HPLC [5–95% of CH_3_CN (0.1% TFA/ H_2_O (0.1%TFA) over 15 min]. (i) Reaction of TCT to replace the first “-Cl” with nucleophile butan-2-amine, followed by addition of 3-methylbutane-2-thiol or 2-phenylethan-1-ol. (ii) Reaction of TCT with nucleophile 3-methylbutane-2-thiol, followed by addition of butan-2-amine or 2-phenylethan-1-ol. (iii) Reaction of TCT with nucleophile 2-phenylethan-1-ol, followed by addition of butan-2-amine or 3-methylbutane-2-thiol.

Encouraged by these results, an attempt was made to explore the third position for aromatic nucleophilic substitution in compounds **4** (NS-MCT), **5** (NO-MCT), and **6** (SO-MCT). As expected, based on the formation of the di-substituted derivatives, the reaction did not proceed to completion even upon subjecting to harsh conditions, except for that of compound **6** (SO-MCT). Therefore, **6** was reacted with butan-2-amine in the presence of DIEA using THF as solvent. Upon addition of amine, the reaction mixture was heated to 75°C for 12 h, and the reaction was monitored using TLC, as well as HPLC. The reaction yielded 23% conversion to **7**. An additional equivalent of DIEA was added and the reaction was allowed to 18 h at 75°C for complete conversion of **6** to afford final product (**7**) as determined by HPLC. All the compounds were well characterized by NMR, HPLC, and LCMS. Figure 7 depicts the progress of the reaction, as monitored by HPLC.

### Theoretical calculations

**Figure 8:**
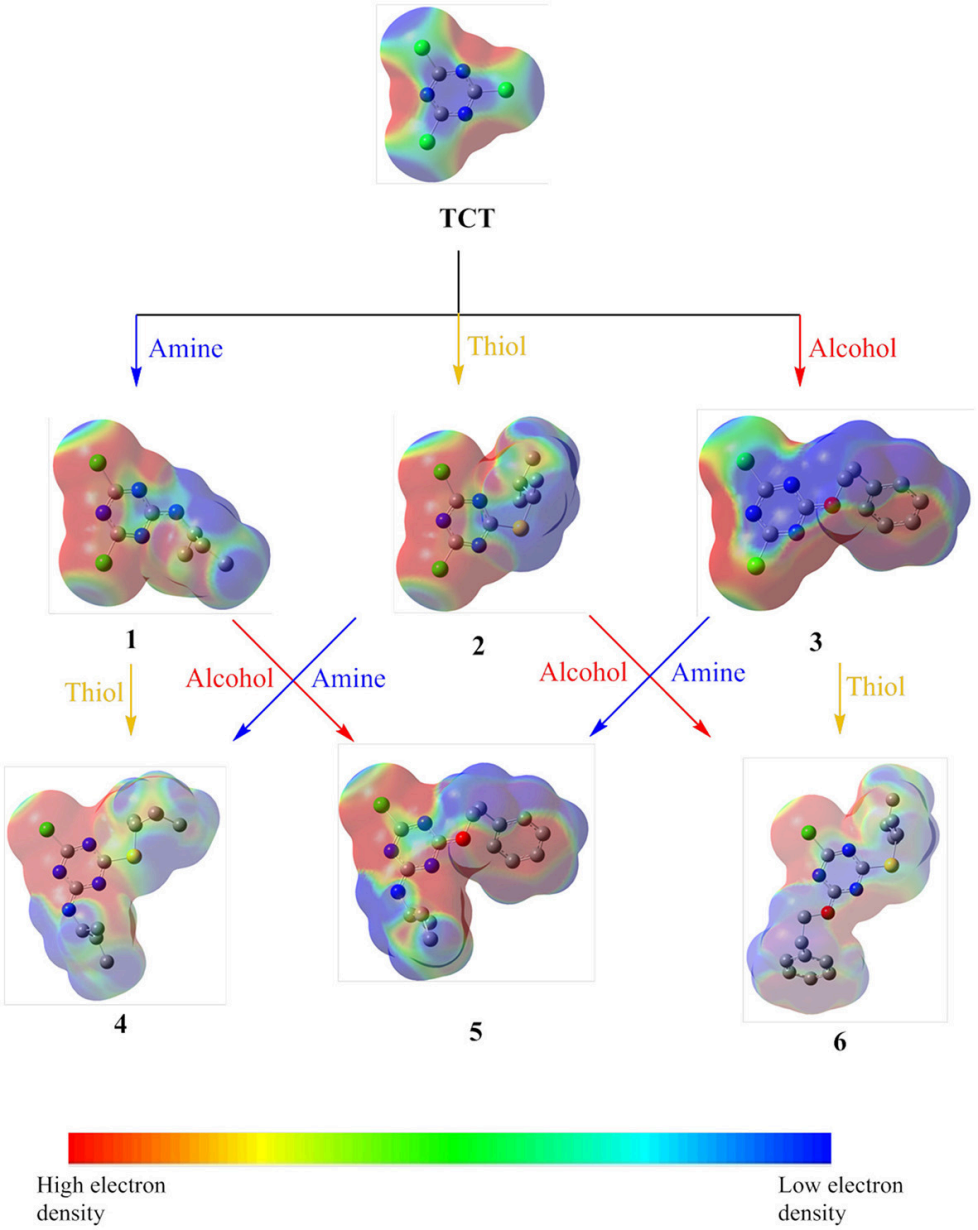
Electrostatic potential maps of TCT with different nucleophiles (amine, thiol and alcohol).

To elucidate the electronic effect on TCT during the nucleophilic substitutions, a density functional theory (DFT) geometry optimization using Gaussian09 program package (Frisch et al., 2009) employing the B3LYP (Becke three parameters Lee–Yang–Parr exchange correlation functional) and the 6-311G++(d,p) basis set were performed in gas phase. B3LYP combines the hybrid exchange functional of Becke (1988) with the gradient-correlation functional of Lee et al. (1988) It can be clearly seen in Figure 8 that TCT is electron-deficient around the carbon linked to the “-Cl” atom. However, upon reaction with different nucleophiles, the electron density changed in derivatives **1**, **2**, and **3**, respectively. Due to the high electronegativity of oxygen, O-DCT (**3**) remained more electron-deficient than the others. This observation is also consistent with the requirement of a low temperature for reaction between different nucleophiles with TCT. Comparison of **4**, **5**, and **6** reveal that the electron density around the carbon directly attached to chlorine in the case of **6** is low with respect to the other derivatives. Hence, the formation of **7** was observed in case of **6** and not with **4** and **5**.

Natural bond orbital (NBO) analysis is another efficient tool for studying hyperconjugative interactions, intermolecular charge transfer, and electron density transfer (EDT), which are fundamentally linked to calculations of atomic charges (Drissi et al., 2015; Md Rauf et al., 2015; Bamba et al., 2016; El-Faham et al., 2016). In the present work, NBO analysis was performed on all the derivatives and comparison was made on the basis of the charge carried by the “-Cl” atom present during the reaction (Supplementary Material).

It is also important to compare the atomic charge present on “-Cl” in all the possible cases (Pearson, 1997). As the greater the charge higher the reactivity toward the nucleophile will be. As seen in Table 1, the first “-Cl” atom in TCT carries an atomic charge of 0.088 units, which makes it a good electrophile and hence reactions proceed faster even at a low temperature of 0°C. However, upon replacement of the first “-Cl” with a nucleophile, as in case of **1**, **2**, and **3**, the decrease of positive charge on “-Cl” was observed. Thus, the reaction requires a higher temperature (room temperature in this case). Upon comparison, **1** has a low atomic charge on both “-Cl” (0.048 and 0.047 units) and hence could be the plausible reason for the absence of further reactivity at room temperature. However, in the case of **2** and **3**, the reactions proceeded smoothly at room temperature, leading to the respective XY-MCT. These findings indicate that amine is not preferable to be the first substituent in case of TCT.

**Table 1:** Shows the electronic charges present on “-Cl” in all the molecules.

**Sl. No**.	**Natural atomic charges on “-Cl” determined using NBO calculations**		
	**Third-Cl**	**Second-Cl**	**First-Cl**
TCT	0.088	0.088	0.088
1	0.048	0.047	–
2	0.066	0.060	–
3	0.067	0.063	–
4	0.023	–	–
5	0.024	–	–
6	0.036	–	–

To further determine the preferential order of nucleophiles for introduction into XY-MCT, we compared the charge present on last “-Cl” in the case of **4**, **5**, and **6**. For **6**, the charge was 0.036, which is higher than that present on **4** and **5**. Hence, the reaction was feasible for **6** but under reflux conditions, affording product **7**. For **4** and **5**, more harsh conditions may be required to make the reaction feasible. These theoretical results provide an insight into the preferential order of incorporation of different nucleophiles in TCT. In this regard, the order was found to be first alcohol (2-phenylethan-1-ol), then thiol (3-methylbutane-2-thiol), and finally amine (butan-2-amine).

## Conclusion

In conclusion, we report the facile ability of TCT to undergo nucleophilic aromatic substitution reaction in presence of distinct nucleophiles (butan-2-amine, 3-methylbutane-2-thiol and 2-phenylethan-1-ol) and the preferential order of subsequent introduction is alcohol, followed by thiol, and finally amine under mild conditions. Theoretical calculations were also performed to explain the experimental results. Both, electron density maps and natural atomic charges were consistent with the experimental results, thereby validating the findings reported.

Taking the definition of *orthogonal chemoselectivity* as “the discrimination among the reactive sites in any order,” it can be concluded that only the double nucleophilic aromatic substitution of S, O on TCT is orthogonally chemoselective, as demonstrated by the two strategies used to obtain **6**. The remaining double substitution and also the triple substitution are only examples of chemoselectivity and not orthogonality. The introduction of amine as the first nucleophile precludes the subsequent incorporation of thiol or alcohol.

However, if the first position is occupied by thiol followed by reaction with amine and alcohol, only amine incorporation proceeds and not alcohol affording **4**. In case of alcohol in first position followed by reaction with amine and thiol, both reaction proceeds to completion affording formation of **5** and **6**. After the double incorporation of nucleophiles into TCT, reactivity of the third position was evaluated and it was confirmed that the preferential order of incorporation of nucleophiles is alcohol > thiol > amine in presence of DIEA and DCM or THF as solvent to afford **7**. The results described herein are expected to be useful in medicinal chemistry for drug design incorporating the *s*-triazine core, as well as for the construction of conjugates of two or three chemical/biological entities.

## Author contributions

Preparation of derivatives and theoretical calculations was done by AS. The manuscript was written with contributions from of all the authors. All authors have given approval to the final version of the manuscript.

### Conflict of interest statement

The authors declare that the research was conducted in the absence of any commercial or financial relationships that could be construed as a potential conflict of interest.

## Footnotes

^1^TCT derivatives has been used mainly as coupling reagents (see Kaminski, 2000 and the recent work of Fraczyk et al., 2018; Kitamura et al., 2018) and as a template in medicinal chemistry (see Nelson et al., 2018 and Sharma et al., 2017).
