# Supplementary material for: Exploring the Orthogonal Chemoselectivity of 2,4,6-Trichloro-1,3,5-Triazine (TCT) as a Trifunctional Linker With Different Nucleophiles: Rules of the Game
Source: Front Chem. 2018 Nov 1;6:516. doi: 10.3389/fchem.2018.00516 (PMC6221914; doi:10.3389/fchem.2018.00516)
Supplement: Supplementary file 1 [file Data_Sheet_1.docx]

Supplementary Material

**Exploring the Orthogonal Chemoselectivity of 2,4,6-Trichloro-1,3,5-triazine (TCT) as a Trifunctional Linker with Different Nucleophiles: Rules of the Game**

**Anamika Sharma,^1^ Ayman El-Faham,^2,3^ Beatriz G. de la Torre,^4^ Fernando Albericio^5,6,7,*^**

*** Correspondence:** Fernando Albericio: albericio@ukzn.ac.za

**Contents**

| **Figure 1** | : | HPLC for compound 1 |
| --- | --- | --- |
| **Figure 2** | : | ^1^H NMR and ^13^C NMR of compound 1 |
| **Figure 3** | : | HPLC for compound 2 |
| **Figure 4** | : | ^1^H NMR and ^13^C NMR of compound 2 |
| **Figure 5** | : | HPLC for compound 3 |
| **Figure 6** | : | ^1^H NMR and ^13^C NMR of compound 3 |
| **Figure 7** | : | HPLC for compound 4 |
| **Figure 8** | : | ^1^H NMR and ^13^C NMR of compound 4 |
| **Figure 9** | : | HPLC for compound 5 |
| **Figure 10** | : | ^1^H NMR and ^13^C NMR of compound 5 |
| **Figure 11** | : | HPLC for compound 6 |
| **Figure 12** | : | ^1^H NMR and ^13^C NMR of compound 6 |
| **Figure 13** | : | HPLC for compound 7 |
| **Figure 14** | : | ^1^H NMR and ^13^C NMR of compound 7 |
| **Figure 15** | : | NBO of TCT |
| **Figure 16** | : | NBO of compound 1 |
| **Figure 17** | : | NBO of compound 2 |
| **Figure 18** | : | NBO of compound 3 |
| **Figure 19** | : | NBO of compound 4 |
| **Figure 20** | : | NBO of compound 5 |
| **Figure 21** | : | NBO of compound 6 |

**Figure 1:** HPLC for compound **1**


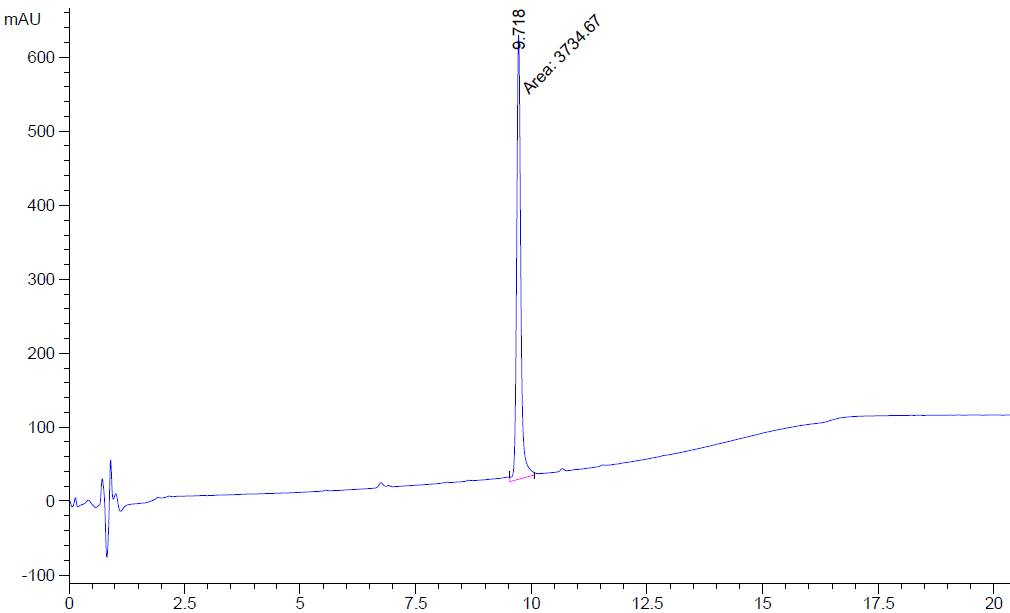


**Figure 2:** ^1^H NMR and ^13^C NMR of compound **1**


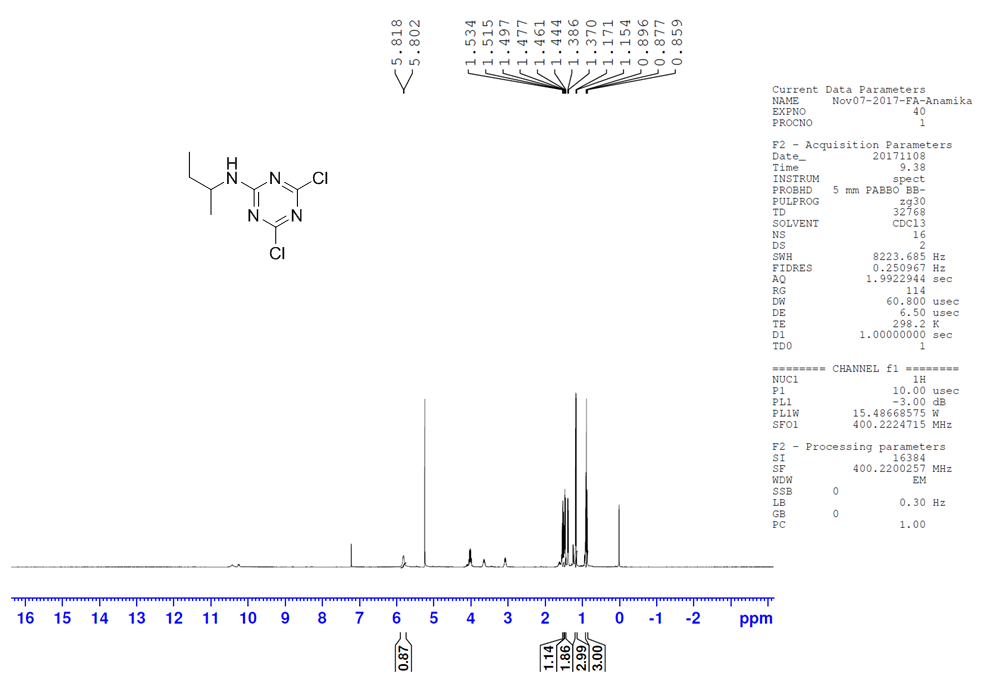


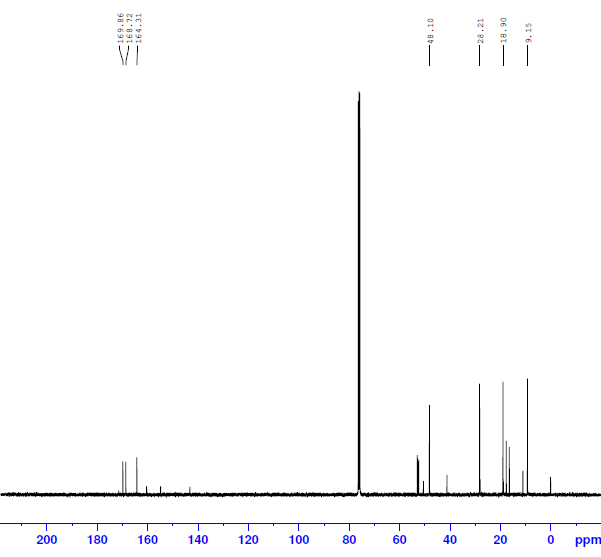


**Figure 3:** HPLC for compound **2**

**
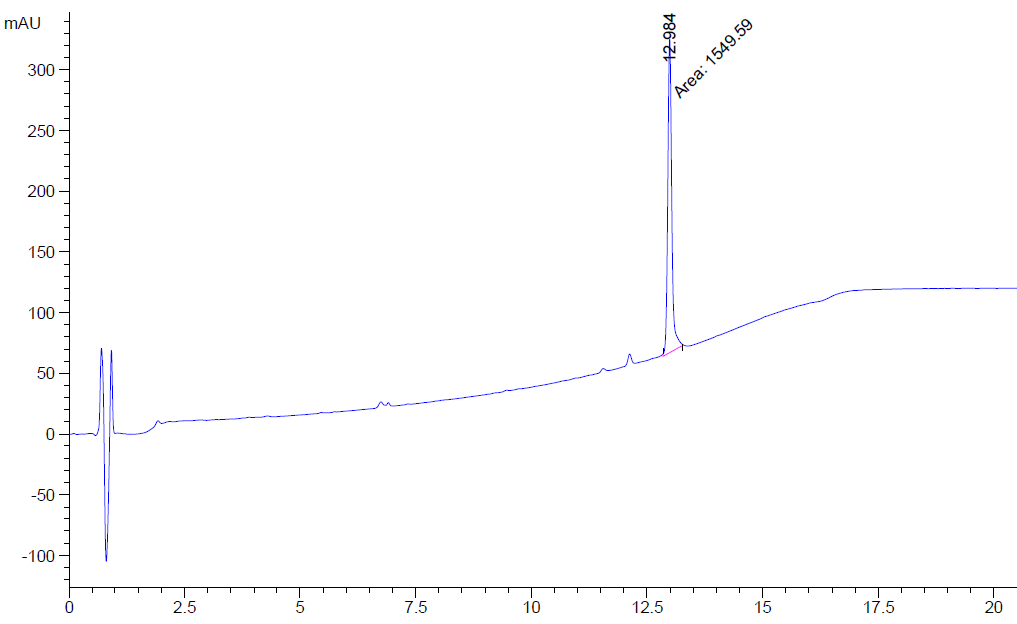
**

**Figure 4:** ^1^H NMR and ^13^C NMR of compound **2**

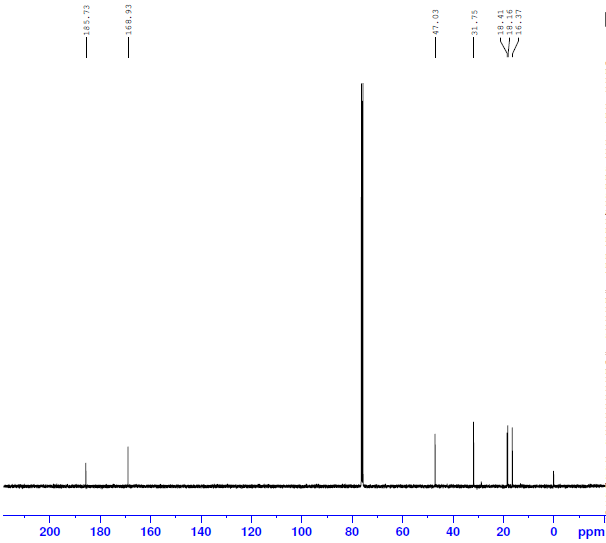


**Figure 5:** HPLC for compound **3**


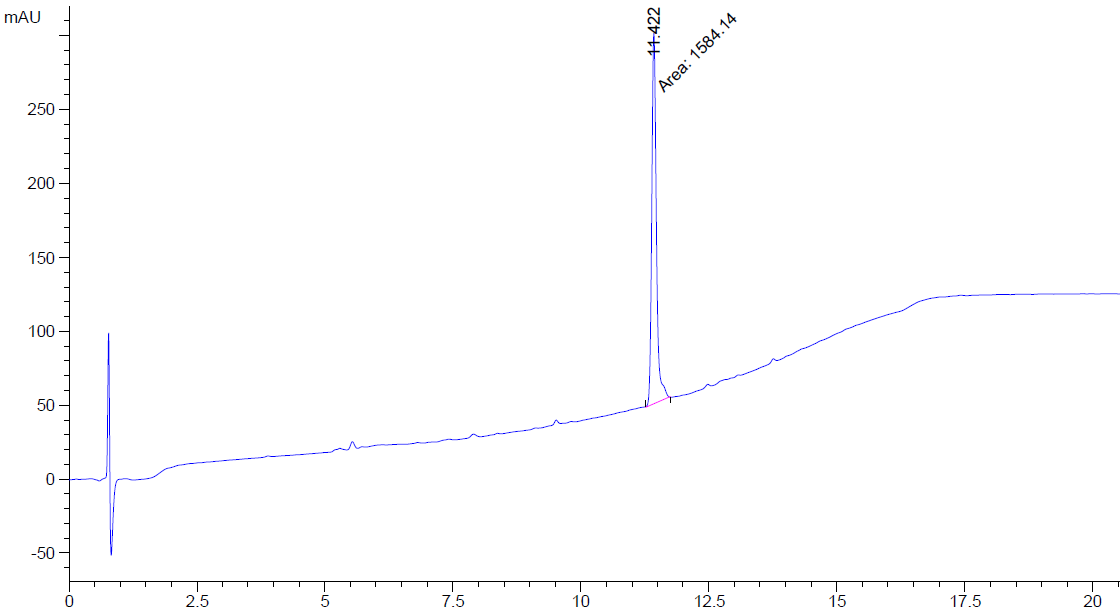


**Figure 6:** ^1^H NMR and ^13^C NMR of compound **3**

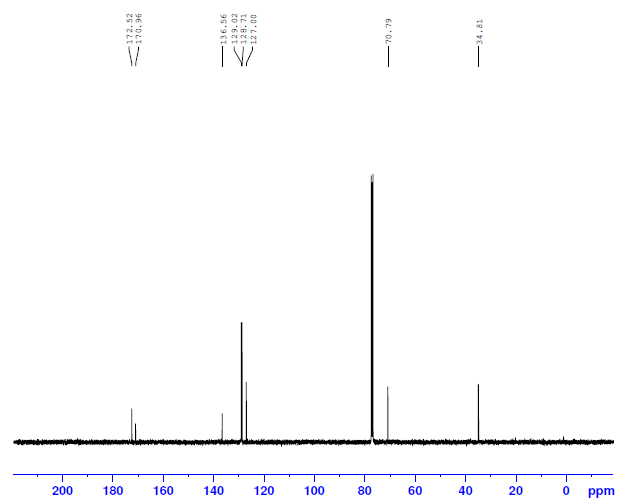


**Figure 7:** HPLC for compound **4**


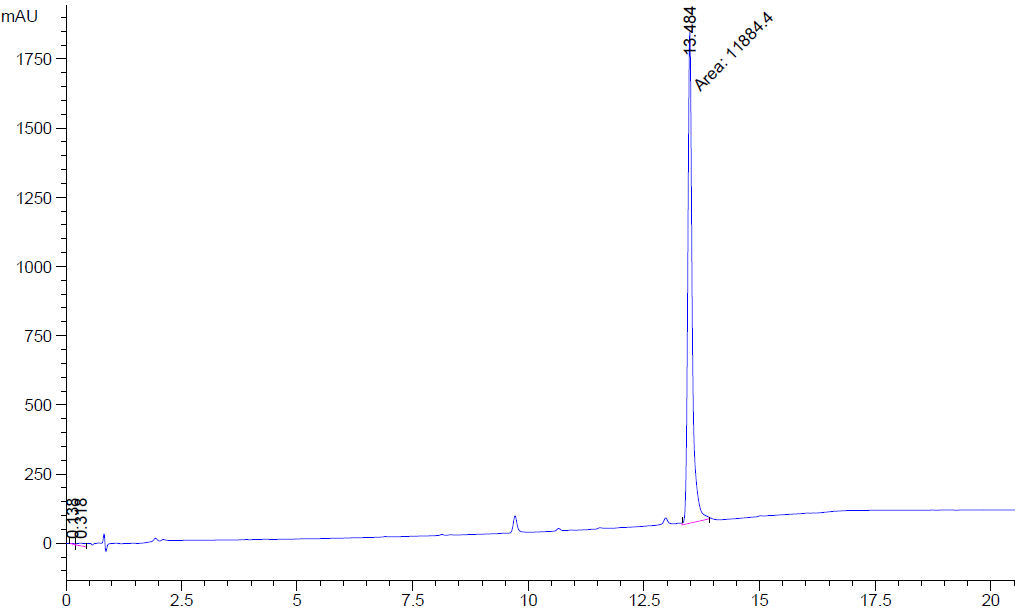


**Figure 8:** ^1^H NMR and ^13^C NMR of compound **4**

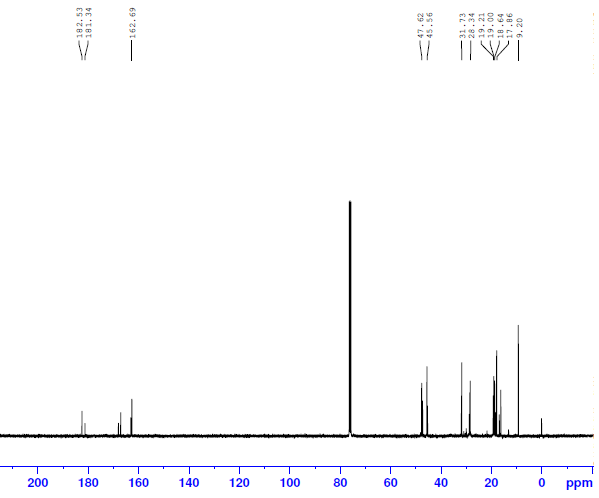


**Figure 9:** HPLC for compound **5**


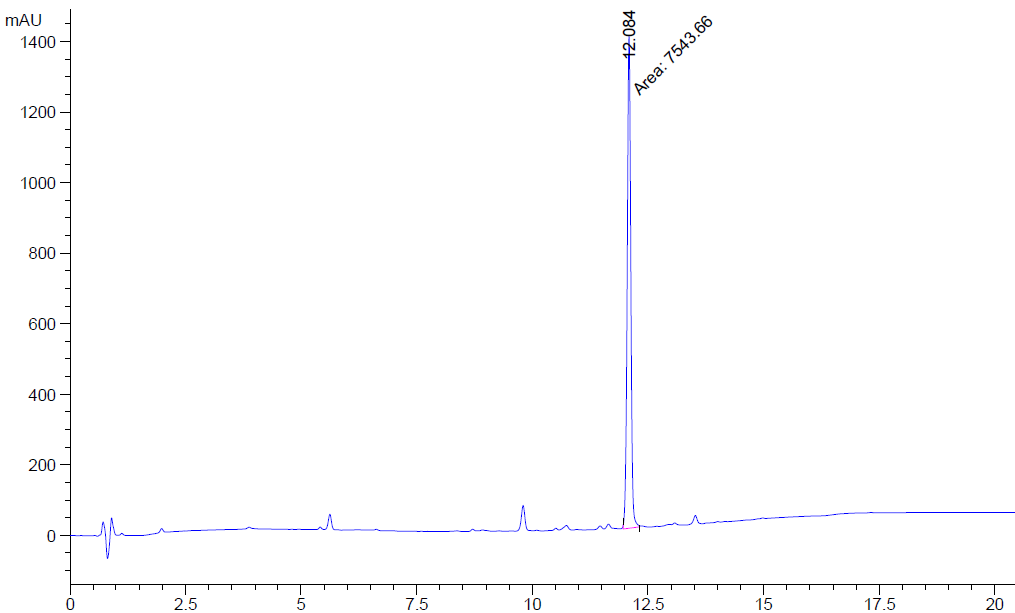


**Figure 10:** ^1^H NMR and ^13^C NMR of compound **5**

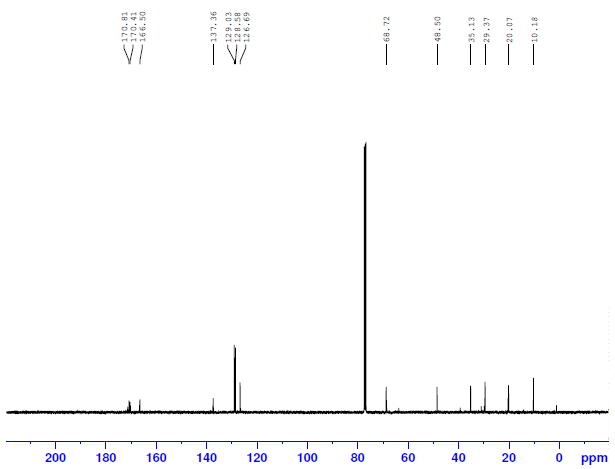


**Figure 11:** HPLC for compound **6**


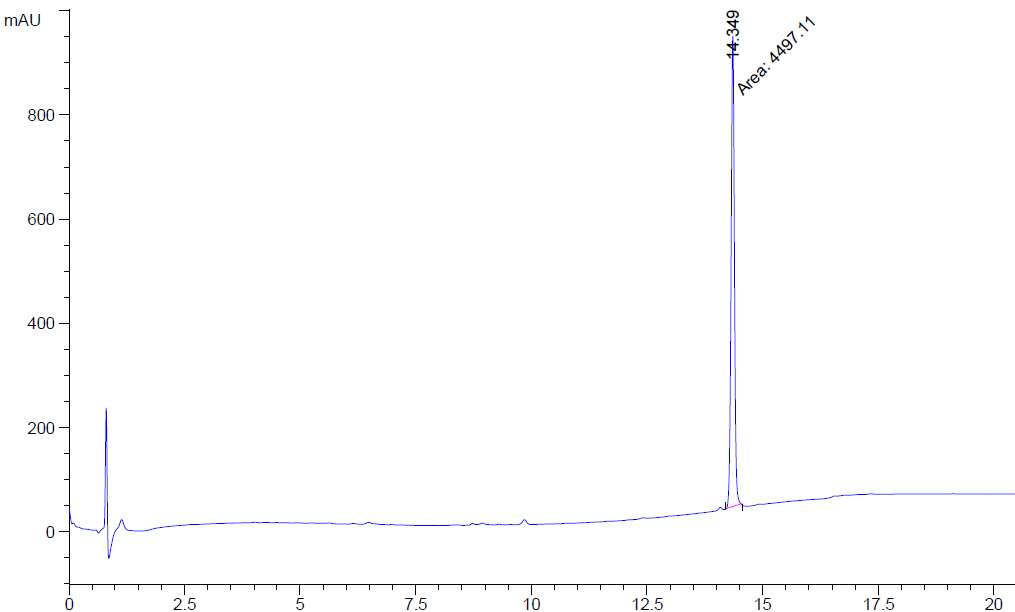


**Figure 12:** ^1^H NMR and ^13^C NMR of compound **6**

**
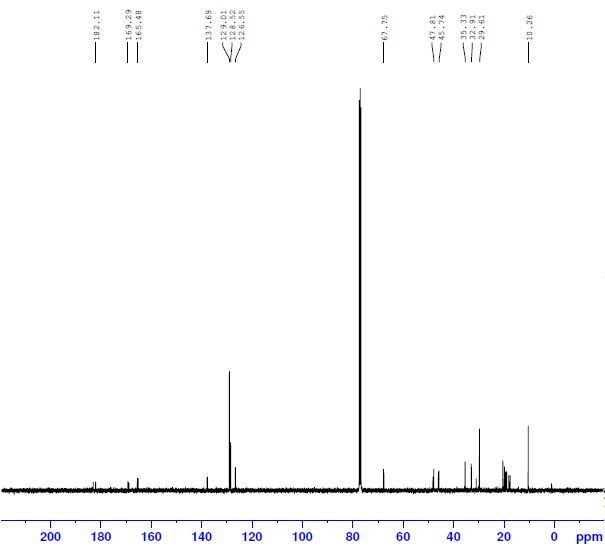
**

**Figure 13:** HPLC for compound **7**


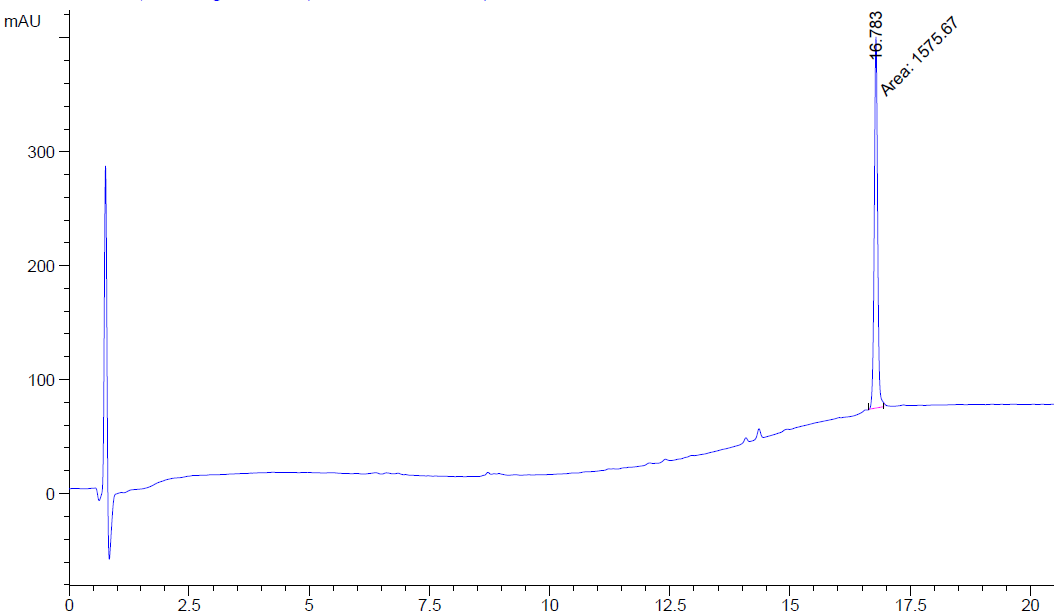


**Figure 14:** ^1^H NMR and ^13^C NMR of compound **7**

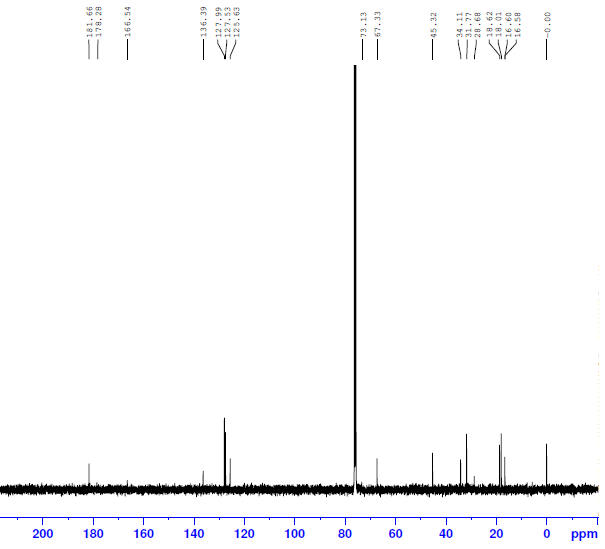


**Figure 15:** NBO of **TCT**


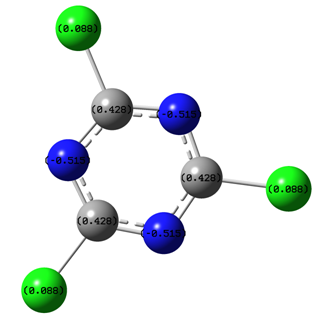


**Figure 16:** NBO of compound **1**

**
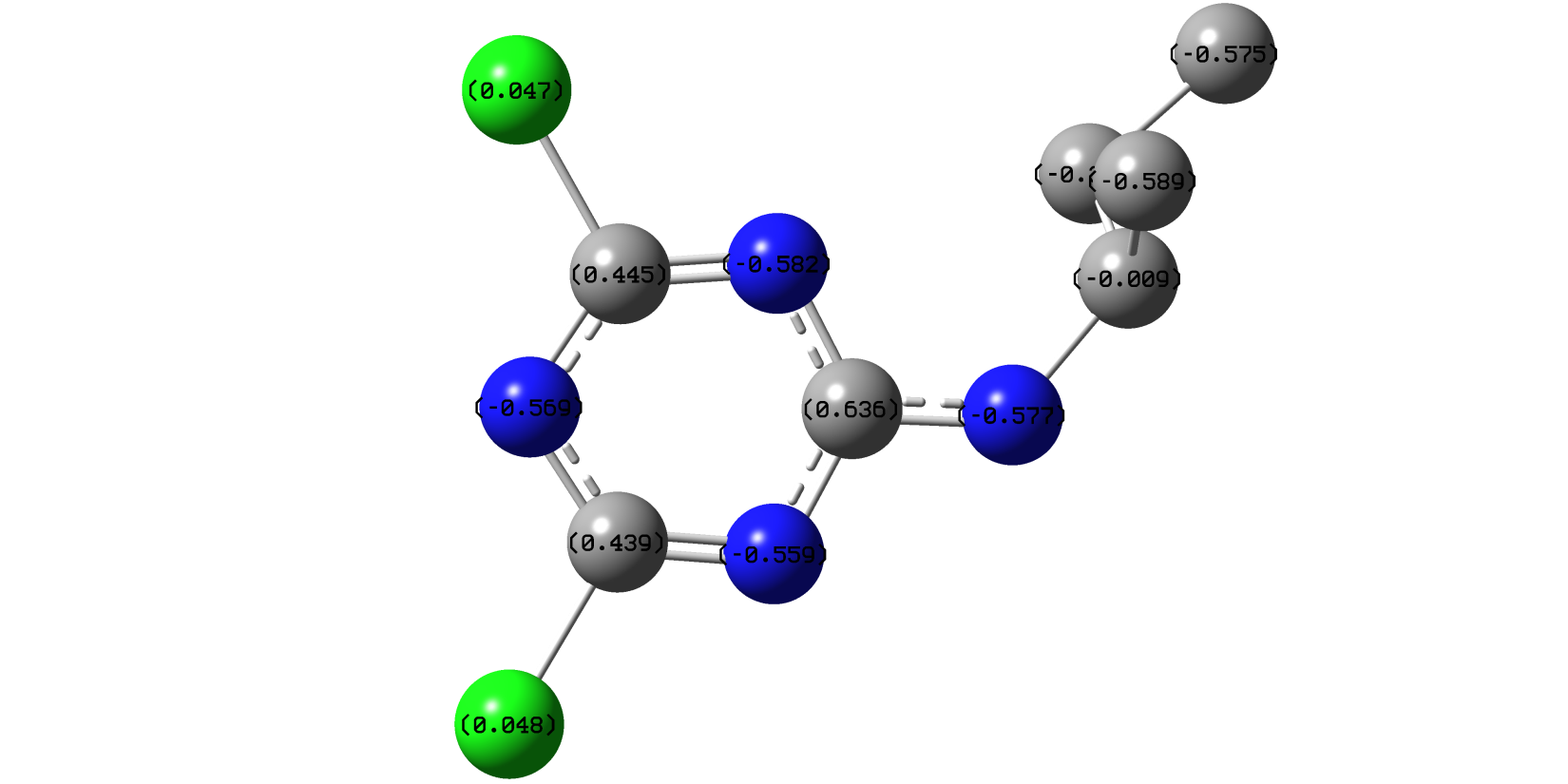
**

**Figure 17:** NBO of compound **2**

**
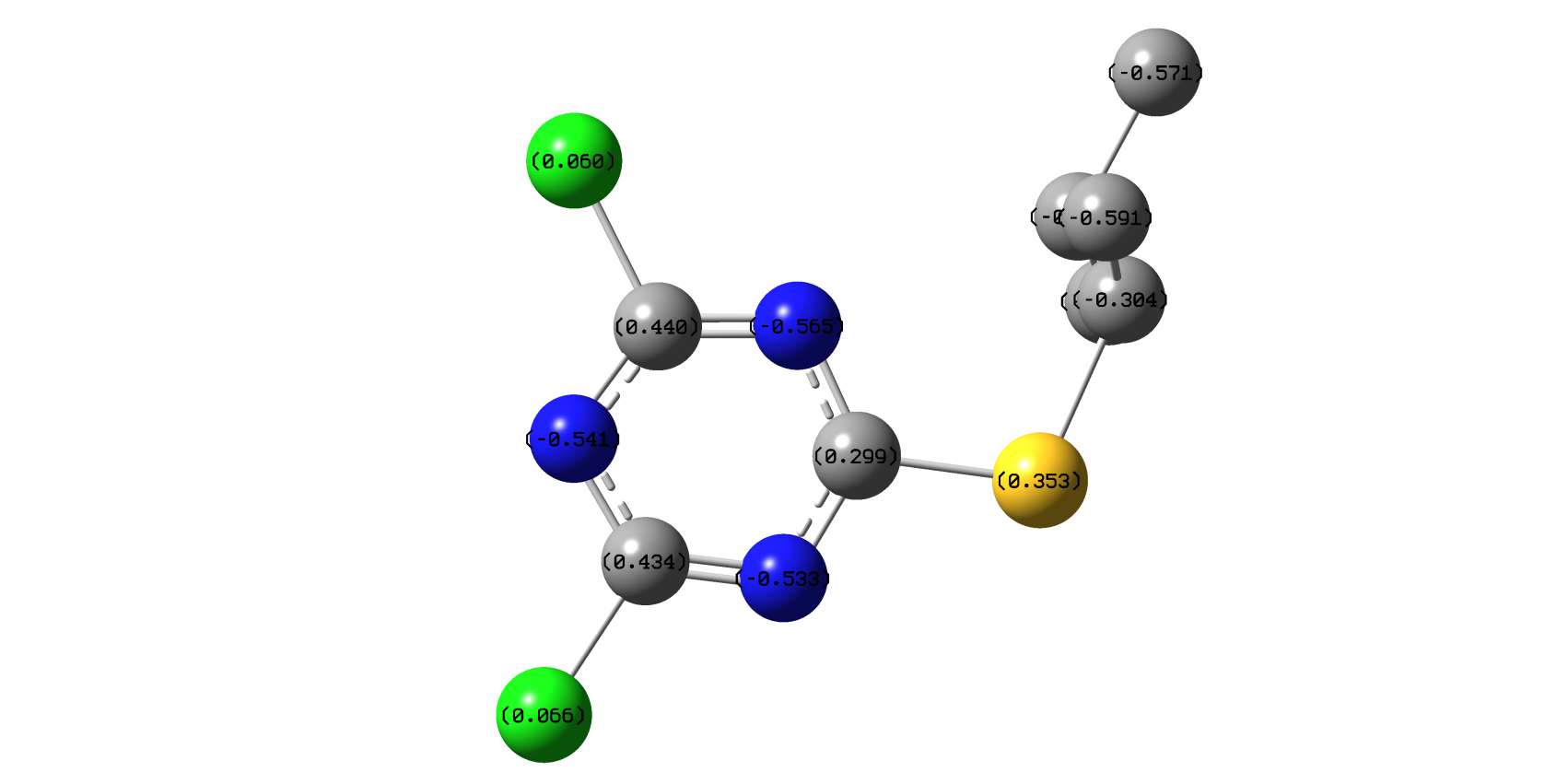
**

**Figure 18:** NBO of compound **3**


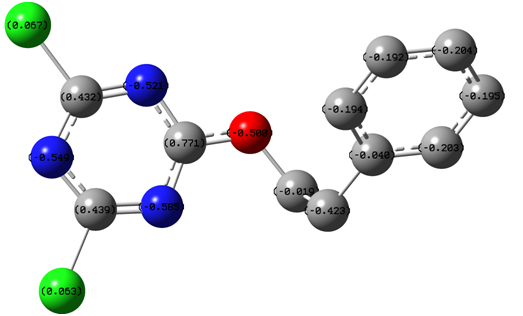


**Figure 19:** NBO of compound **4**

**
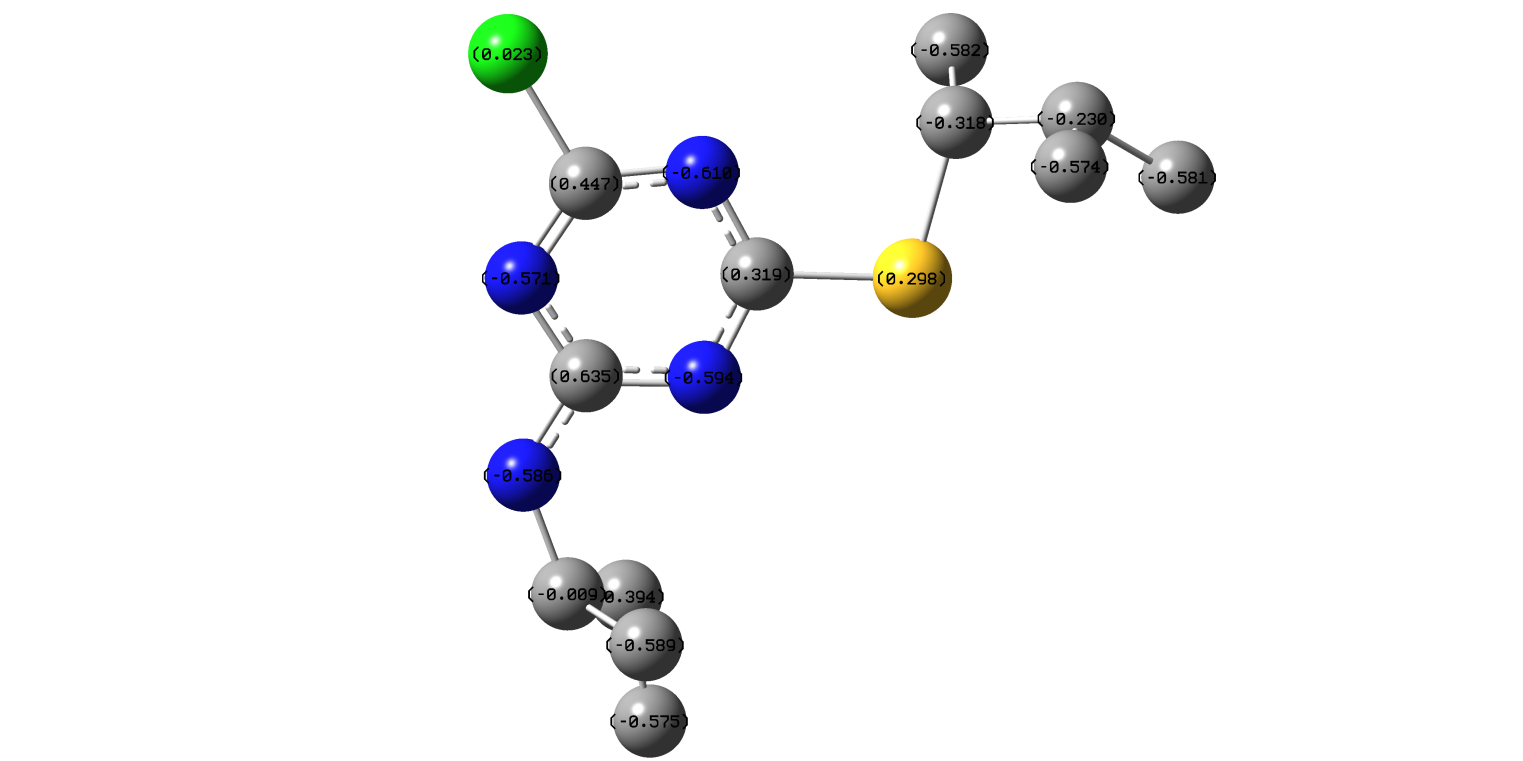
**

**Figure 20:** NBO of compound **5**

**
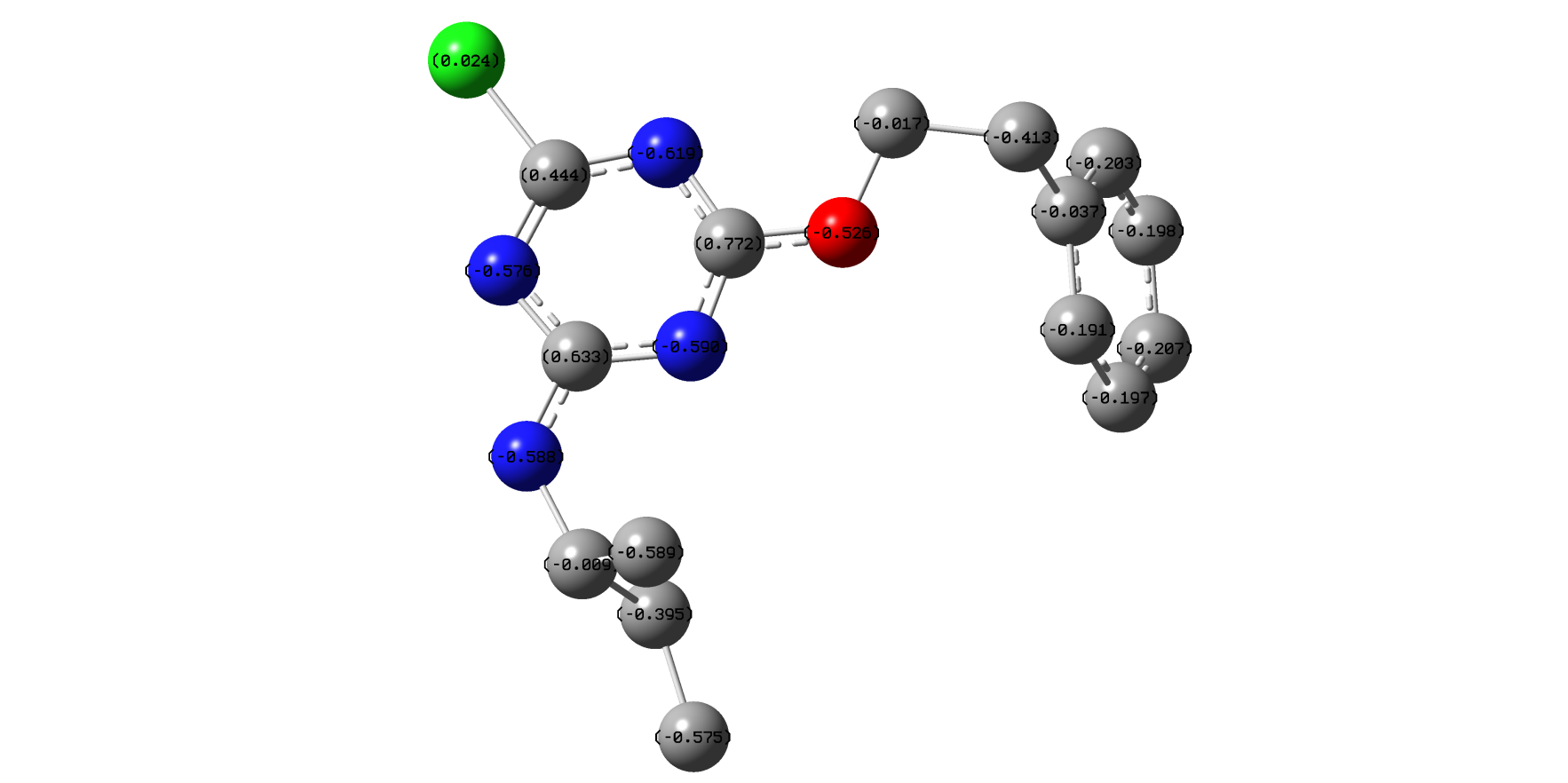
**

**Figure 21:** NBO of compound **6**

**
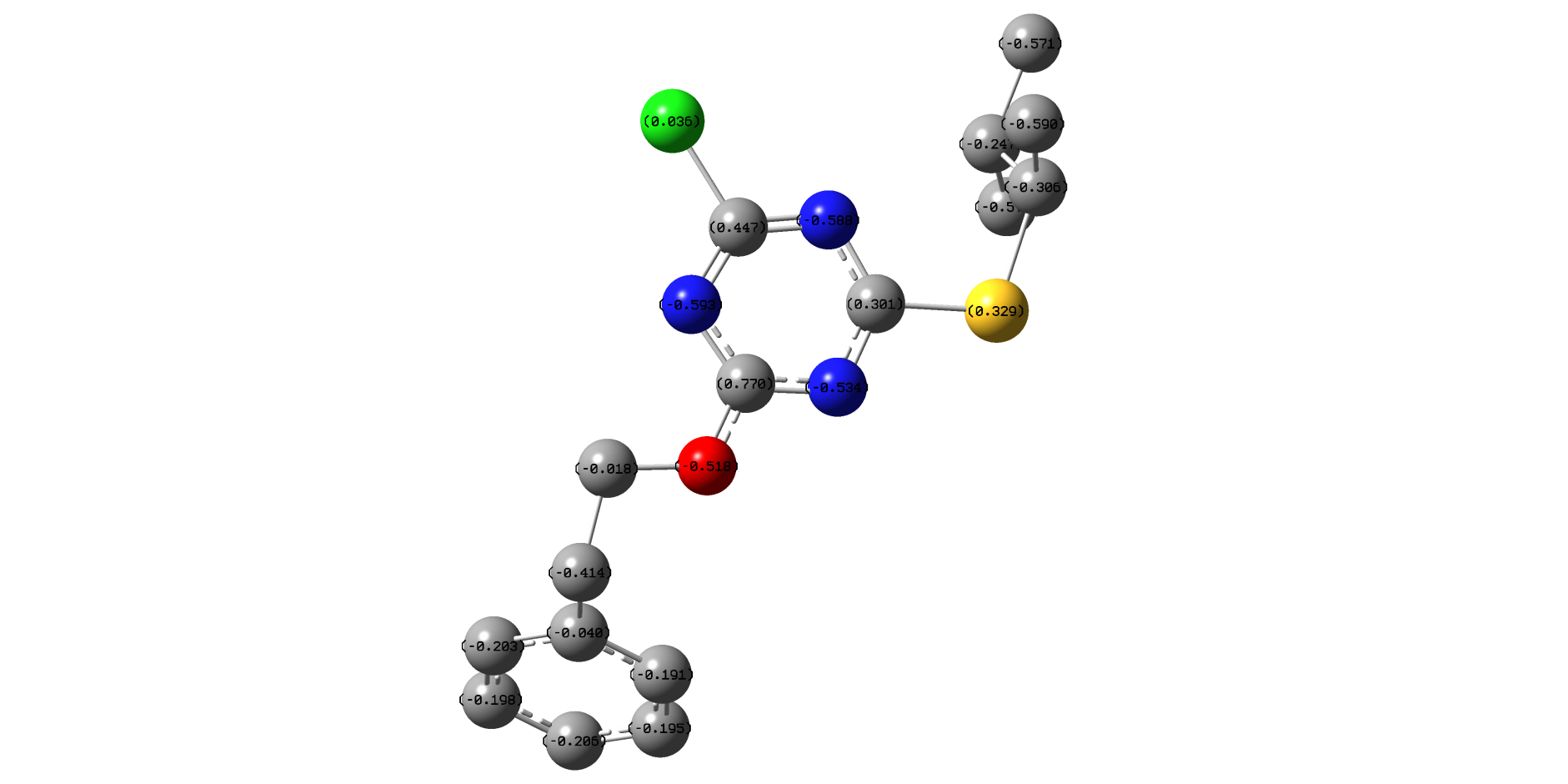
**
